# The factors and motivations behind United Kingdom chiropractic professional association membership: a survey of the Welsh Institute of Chiropractic Alumni

**DOI:** 10.1186/s12998-016-0115-x

**Published:** 2016-09-12

**Authors:** Sheena E. Wotherspoon, Peter W. McCarthy

**Affiliations:** University of South Wales, Pontypridd, Mid Glamorgan, Wales, CF37 1DL UK

**Keywords:** Chiropractic, Professional associations, Alumni, Membership, Organisations, Motivation

## Abstract

**Background:**

There are many professional associations representing chiropractors and chiropractic in the United Kingdom (UK). Each has its unique selling points (USPs) and chiropractors can choose to join as many as they like; however, cost of membership has to be weighed against perceived benefits. The predictors of UK chiropractic association membership and motivational factors to join these associations, have not formally been identified. This research study aimed to identify some of the factors and motivations in Welsh Institute of Chiropractic (WIOC) Alumni regarding their decision to join (or not) a UK chiropractic professional association.

**Methods:**

An online survey instrument, comprising 23 questions, was administered from November-December 2015 via a link announced on ‘The WIOC Alumni’ Facebook group (*N* = 655), the active platform for the WIOC Alumni Organisation.

**Results:**

One hundred forty-eight respondents (approximately 22.6 % of ‘The WIOC Alumni’ Facebook group membership) completed the survey. Ten factors were reported to be important in decision making: ‘promoting public awareness of chiropractic’ (91.2 %), ‘access to professional indemnity insurance’ (89.2 %), ‘overall professionalism of the association’ (87.2 %), ‘the identity of the association’ (77.7 %), ‘positive attitude to research’ (77.0 %), ‘workplace support and advice’ (68.9 %), ‘access to events \ courses \ seminars’ (64.2 %), ‘Continuing Professional Development (CPD) activities’ (62.2 %), ‘cost of membership’ (59.5 %) and ‘addresses my area of interest’ (56.1 %). ‘Many of my friends have joined’ (71.6 %) was considered unimportant, whereas ‘Lobbying: Influencing policy’ and ‘career development’ were considered important by almost twice as many as those that consider them unimportant (45.3 %: 25.7 % and 43.9 %: 27.0 % respectively), ‘requirement of employment’ and ‘associations newsletter’ were seen as unimportant by roughly twice as many as those considering them important (44.6 %: 28.4 % and 35.8 %: 28.4 % respectively). Should it become an option, almost 71 % of respondents would support the unification of the four main UK chiropractic associations, (the British, McTimoney, Scottish and United, Chiropractic Associations: BCA MCA, SCA and UCA, respectively).

**Conclusions:**

Several factors have an important effect on motivations to join UK chiropractic professional associations. Further research is required to determine if this is WIOC specific or can be extrapolated more generally.

**Electronic supplementary material:**

The online version of this article (doi:10.1186/s12998-016-0115-x) contains supplementary material, which is available to authorized users.

## Background

A professional association can be defined as a body of persons engaged in the same profession. Its uses include controlling entry into the profession, maintaining standards, and representing the profession in discussions with other bodies [1]. Professional associations are generally considered to be bodies which give a form of identification and organisation within fields of professional practice. These associations facilitate field knowledge, provide normative frameworks for practice and serve as catalysts for change [2, 3]. For the purposes of this research, ‘professional association’ will be used as an overarching umbrella term encompassing professional membership bodies, research bodies, the Associations and other organisations that function to facilitate development of chiropractors and chiropractic.

The literature reveals a general consensus that the establishment of a professional association is an essential step for achieving and maintaining a professional status and indeed a profession [4–9]. The credibility of a profession is generally considered to be a measure of both the vitality and credibility of its associations [7]. A stronger, more engaged membership serves to benefit not only the organisation, in advancing the profession, but also can positively impact on the professional development of the membership [5, 7].

### UK chiropractic professional associations

It has been acknowledged in the literature that the chiropractic profession has been plagued throughout its history by internal ideologically-based strife [10–14]. Effects of this can be seen in the UK, where a range of chiropractic professional associations compete with each other for members, however, the theme of being stronger together (unity) is apparent [12, 15–17]. At the British Association of Chiropractic Students (BACS) 2015 conference [18], the leaders of the main UK chiropractic associations, the British, McTimoney, and United, (the Scottish gave their apologies) Chiropractic Associations (BCA MCA, UCA, and SCA respectively), participated in an inter-association debate, discussing ‘unity’. These associations agreed to the principle of unification of the four main UK chiropractic associations (BCA, MCA, SCA and UCA) sometime in the future [18]. How, when and if this will ever be achieved is currently undecided. Table 1 provides an overview of the main chiropractic professional associations within the UK, including, for the purpose of this research, the professional association ‘type’.

**Table 1:** UK chiropractic professional associations

Name	Type	Description	Web address
British Chiropractic Association (BCA)	Association (acting like a Trade Union)	Founded in 1925 with a membership comprising over 50 % of the UK’s registered chiropractors [38].	http://www.chiropractic-uk.co.uk/.
McTimoney Chiropractic Association (MCA)	Association (acting like a Trade Union)	Established for over 35 years with over 550 members in the UK and Eire [39].	http://www.mctimoney-chiropractic.org/
Scottish Chiropractic Association (SCA)	Association (acting like a Trade Union)	Formed in 1979 and has more than 60 members practising in Scotland and over 120 associated members located in England, Wales, Northern Ireland and various other countries around the world [40].	http://www.sca-chiropractic.org/
United Chiropractic Association (UCA)	Association (acting like a Trade Union)	The UCA is the youngest chiropractic association in the UK and is committed to protecting and advancing the principles of vitalistic chiropractic, with a membership growing steadily since 2000 [41].	http://www.united-chiropractic.org/
Chiropractic Research Council (CRC)	Research body	Established in 2013 to provide leadership and unity in chiropractic research, aiming to increase the chiropractic evidence base, by promoting research, increasing the research capacity and supporting researchers [42].	http://www.crc-uk.org/
Royal College of Chiropractors (RCC) (formerly College of Chiropractors)	Professional membership body	A Royal Chartered academic membership organisation with over 2800 members worldwide. Conceived during 1997 and incorporated in 1998 as an independent body to develop, encourage and maintain the highest possible standards of chiropractic practice for the benefit of patients [43].	http://rcc-uk.org/
Alliance of UK Chiropractors (AUKC)	‘Others’: an option for alternative responses	An affiliation of professional organisations [44]. In 2010 the UCA, SCA, and MCA formed the AUKC whose vision is “to create a vitalistic, Chiropractic model of health and well-being for families in the UK by providing the distinct elements offered by Chiropractic as a healthcare profession predicated upon its philosophy, science and art.” [45].	
British Veterinary Chiropractic Association (BVCA)	‘Others’: an option for alternative responses	A national non-profit organisation dedicated to promoting excellence in the field of Veterinary Chiropractic in the UK. There are approximately 50 members listed [46].	http://bvca-uk.org/

This table contains the name and website details (where available) along with aspects of their individual Unique Selling Points

Many professional associations representing chiropractors and chiropractic exist in the United Kingdom (UK). Each purports to have its own unique selling points (USPs) and chiropractors can choose to be a member of as many as they like. However, in the current economic climate cost of membership has to be weighed against perceived benefits. There appear to be many benefits and, thus, motivators to joining a professional association [19]. The five main motivators for membership relate to perception of: the cost to benefit ratio, the organisation’s role as an advocate for professional issues, the perception that the organisation’s operation is fair and that members’ views are taken into account, value of the annual meeting, and that professional identify and status are enhanced by membership [19]. A variety of other factors have also been identified including, but not limited to: journal quality, networking opportunities and access to professional liability insurance, developing new skills and competencies, professional/career development, Continuing Professional Development (CPD) activities, mentoring opportunities, keeping abreast of contemporary topics through newsletters and or associated journals [4, 20].

At present, little data exists regarding what motivates United Kingdom (UK) registered chiropractors and chiropractic students to join UK chiropractic professional associations. Similarly, predictors of UK chiropractic professional association membership have not formally been identified. As with any professional association membership other important considerations include the alignment with an appropriate professional identity and training [4] and a variety of personal and environmental forces (including economics) which affect degree of active participation in, and donation to, professional associations [2, 21]. In all of these areas, there has been little chiropractic specific research published to date.

In some professional associations there is no choice element in relation to membership. One such example is membership of the General Chiropractic Council (GCC): in order to practice as a chiropractor in the UK, membership of the GCC is mandatory. Therefore, along with educational institutional alumni, where membership is automatic, these are excluded as professional associations for the purposes of this study. Only those UK chiropractic professional associations where membership is discretionary have been considered in this research.

This research study was conducted through a bespoke online survey to determine some of the factors and motivations of UK chiropractic professional association membership in Welsh Institute of Chiropractic (WIOC) Alumni regarding their decision to join (or not) a UK chiropractic professional association. A question was also posed regarding supporting the unification of the four main UK chiropractic associations, should it become an option sometime in the future.

## Methods

### Survey development

The survey instrument was developed by the authors between June and November 2015, with consideration of previously implemented instruments that examined motivations and predictors of membership [2, 5–7, 22, 23]. Questions were further developed using published literature [4, 8, 19, 24–26].

The survey instrument was designed taking into account best design principles and methodology including formatting content and presentation [27–29] and further designed in a manner to combat survey fatigue [27, 30].

It was deemed that the question of motivation be asked of a single group with a common educational background initially, in order to reduce the impact of variables associated with the more subtle educational influences across institutions. ‘The WIOC Alumni’ Facebook group [31], the active platform for the WIOC Alumni Organisation, was considered the most appropriate method of contacting a reasonably large proportion of the WIOC graduates. (Please note WIOC is the base for the chiropractic undergraduate programme at the University of South Wales: previously the University of Glamorgan until 2013).

Face validity was performed using WIOC faculty members and graduates from the 2015, 2006, and 2005 cohorts.

The final survey instrument totalled 23 questions, 21 of which were mandatory and two optional, covering demographics, memberships, factors and motivations. One question related to unification of the four main UK chiropractic associations: British Chiropractic Association, McTimoney Chiropractic Association, Scottish Chiropractic Association and United Chiropractic Association (BCA, MCA, SCA and UCA, respectively). The survey was constructed to be completed within five - ten minutes. Question types consisted of: multiple choice (*n* = 16), Likert scale (5-point: *n* = 1), free text (*n* = 2), selection list (*n* = 1) and multiple answer (*n* = 3). A copy of the final survey instrument can be found as an Additional file 1. 

### Survey administration and data management

The online instrument was delivered using Bristol Online Surveys (BOS; University of Bristol) with a link to the survey uploaded to ‘The WIOC Alumni’ Facebook group, the active platform for the WIOC Alumni Organisation.

Inclusion criteria: any member of ‘The WIOC Alumni’ Facebook group (not necessarily a WIOC graduate).

Exclusion criteria: non-chiropractic educated member of ‘The WIOC Alumni’ Facebook group.

The survey ran for a one month period from Wednesday the 4^th^ November 2015 through to the close of Friday the 4^th^ December 2015. Five formal reminders were issued during this period, in line with the periodicity of follow-up mailings noted by Sánchez-Fernández, Muñoz-Leiva and Montoro-Ríos [32].

All data was collected and stored anonymously via BOS. No individuals were identified in this research study and consent to take part was implied by completion and submission of the survey.

### Data analysis

Results were downloaded from BOS directly into SPSS (version 22, IBM) for statistical analysis. Normality tests were performed confirming the data was not normally distributed.

Measures of the factors and motivations were derived by collapsing both the lower and upper Likert scale 5-point categories to clarify the ‘unimportant’ and ‘important’ relationships for analysis. Thus the 5-point Likert scale became a 3-point Likert scale of ‘unimportant’, ‘moderately important’ and ‘important’.

## Results

### Proportion of respondents

One hundred and forty eight members of the total recorded WIOC Alumni Facebook membership of 655 (22.6 %, as of 4^th^ December 2015) returned fully completed surveys. Of these, four respondents were non-WIOC graduates and 20 respondents were final year WIOC students.

The WIOC has graduated 917 chiropractors from its launch in 1997, (with inaugural graduation year 2001), up to the end of the 2015 year, with 447 being male and 470 being female: additionally, there have been six known deaths of WIOC graduates. Graduated respondents ranged from the 2001 (0.7 %) to the 2015 (6.8 %) cohorts (graduated cohorts response rate ranged from 0.7–11.5 %), with a median of 6.8 % and average of 5.6 %. The 2016 final year student cohort represented a 12.8 % response. In total 71 males and 77 females completed the survey.

Following making adjustments, such as removing student status respondents and those who graduated from educational institutions other than WIOC, a total of 124 WIOC graduates had successfully completed this survey representing a 13.6 % actual response rate of known living WIOC graduates.

### Membership of associations

Table 2 shows responses regarding student and graduate membership of the UK chiropractic professional associations, (multiple responses could be given). On graduation, 88.8 % were a member of a UK chiropractic professional association: with the BCA (approximately 50 %), RCC (approximately 20 %) and UCA (approximately 15 %) being the three main target UK chiropractic professional associations of WIOC Alumni; both when a student and upon graduation.

**Table 2:** UK chiropractic professional association membership in the WIOC Alumni

	Membership when a student				Membership upon graduation			
	n	%	Male	Female	n	%	Male	Female
Currently not a member of any	NA	NA	NA	NA	11	4.9	6	5
I was/have never been a student member	32	14.2	19	13	NA	NA	NA	NA
As yet undecided	NA	NA	NA	NA	9	4	3	6
Have/Will not join any	NA	NA	NA	NA	5	2.2	3	2
BCA	103	45.6	47	56	101	45.1	46	55
MCA	0	0.0	0	0	0	0.0	0	0
SCA	2	0.9	0	2	9	4.0	5	4
UCA	42	18.6	18	24	33	14.7	16	17
RCC	45	19.9	14	31	52	23.2	18	34
CRC	NA	NA	NA	NA	2	0.9	1	1
Others	2^a^	0.9	1	1	2^b^	0.9	1	1
Total	226	100.0	99	127	224	100.0	99	125

The questions above allowed for multiple answers: i.e. more than one box could be selected. The column n relates to the total number of respondents in that category (% response converts this to a percentage of the total population), NA indicates a response option that was not applicable. Other abbreviations: BCA, British Chiropractic Association; MCA, McTimoney Chiropractic Association; SCA, Scottish Chiropractic Association; UCA, United Chiropractic Association; RCC, Royal College of Chiropractors; CRC, Chiropractic Research Council. ^a^One pre-2000 AECC graduate in a time when student memberships did not exist and one FICS student member. ^b^One EAC member and one chiropractor who is non-practicing

The majority of respondents reported the following factors to be important in their decision making: ‘promoting public awareness of chiropractic’ (91.2 %), ‘access to professional indemnity insurance’ (89.2 %), ‘overall professionalism of the association’ (87.2 %), ‘the identity of the association’ (77.7 %), ‘positive attitude to research’ (77.0 %), ‘workplace support and advice’ (68.9 %), ‘access to events\courses\seminars’ (64.2 %), ‘Continuing Professional Development (CPD) activities’ (62.2 %), ‘cost of membership’ (59.5 %) and ‘addresses my area of interest’ (56.1 %). ‘Lobbying: Influencing policy’ and ‘career development’ were considered important by almost twice as many as those that consider them unimportant (45.3 %: 25.7 % and 43.9 %: 27.0 % respectively: see Table 3 for more details). In contrast, ‘many of my friends have joined’ was considered unimportant (71.6 %), ‘requirement of employment’ and ‘associations newsletter’ were seen as unimportant by roughly twice as many as those considering them important (44.6 %: 28.4 % and 35.8 %: 28.4 % respectively).

**Table 3:** Factors influencing WIOC Alumni to join UK chiropractic professional associations

Factor	Unimportant		Moderately important		Important	
	N	%	N	%	N	%
Promoting public awareness of chiropractic	7	4.7	6	4.1	135	91.2
Access to professional indemnity insurance	4	2.7	12	8.1	132	89.2
Overall professionalism of the association	7	4.7	12	8.1	129	87.2
The identity of the association	12	8.1	21	14.2	115	77.7
Positive attitude to research	8	5.4	26	17.6	114	77.0
Workplace support and advice	16	10.8	30	20.3	102	68.9
Access to events \ courses \ seminars	25	16.9	28	18.9	95	64.2
Continuing Professional Development (CPD) activities	27	18.2	29	19.6	92	62.2
Cost of membership	15	10.1	45	30.4	88	59.5
Addresses my area of interest	17	11.5	48	32.4	83	56.1
Lobbying: Influencing policy	38	25.7	43	29.1	67	45.3
Career development	40	27.0	43	29.1	65	43.9
Associations newsletter	53	35.8	53	35.8	42	28.4
Requirement of employment	66	44.6	40	27.0	42	28.4
Many of my friends have joined	106	71.6	23	15.5	19	12.8

The perceived influence of those factors present in the questionnaire in relation to the respondent’s decision to join a chiropractic professional association. Total number of respondents (148) was the same for each option presented, N indicates the number (and its relative percentage [%]) responding in each component

Additional free text factors included: philosophy and ethos, style of practice and prescribing rights.

Two thirds (66.9 %) of respondents agree that membership in any professional association is one of the hallmarks of a professional person; 33.1 % disagreed. Over three quarters (78.4 %) of the respondents agreed that chiropractic professional associations increase the visibility of the chiropractic profession within the UK; 21.6 % disagreed. If a suitable company providing indemnity insurance was available, just over one third (35.1 %) of respondents would forgo membership of the existing UK chiropractic associations. (See Table 4 for a further breakdown of the above results).

**Table 4:** Additional membership factors and motivators of the whole cohort

Variable	Proportion %	N
Membership is a hallmark of professional person		
Agree	66.9	99
Disagree	33.1	49
Chiropractic professional associations increase the visibility of the chiropractic profession within the UK		
Yes	78.4	116
No	21.6	32
Forgo membership of the existing UK chiropractic associations if a suitable company providing indemnity insurance is available?		
Yes	35.1	52
No	64.9	96

Proportion of the respondents (as a percentage and total number [N]) to the questions relating to additional factors regarding those aspects motivating the chiropractor to join or leave an association: common to all associations

Excluding membership as a student, nearly one third (31.1 %) of respondents have resigned or not renewed membership of a UK chiropractic association. The reasons given in free text included: cost, perceived poor value for money, lack of perceived benefit, philosophy, high insurance, limited access to seminars and CPD, disagreed with aspects of promotion of the profession, identity, lack of support, due to clinic requirements, poor service, conference focus, politics and relocating.

**Fig. 1:**
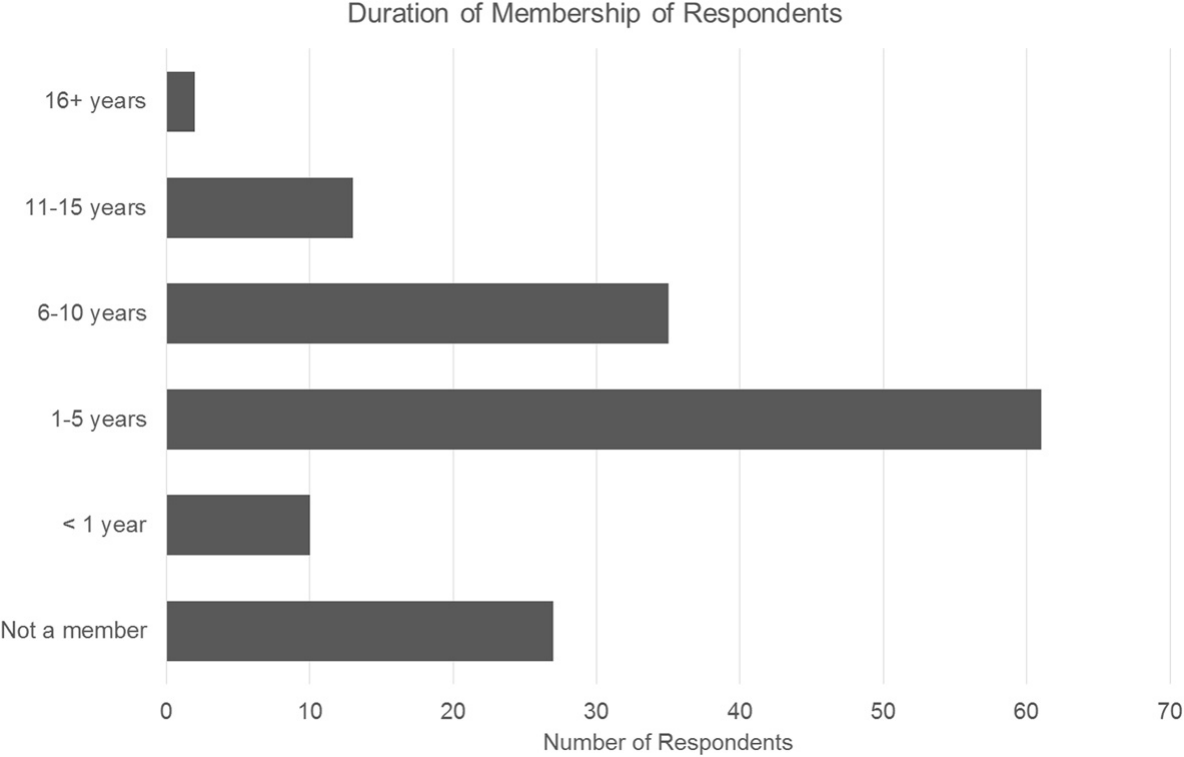
Duration of membership. Histogram showing data indicating the length of time (excluding any student membership) that graduated WIOC alumni chiropractors reported having been a member of a UK chiropractic association

Approximately two fifths of respondents had been a member of a UK chiropractic professional association for 1–5 years (see Fig. 1).

Almost 71 % of respondents would support the unification of the four main chiropractic associations, (BCA, MCA, SCA and UCA), should it become an option sometime in the future. 29 % would not support this.

## Discussion

An aspect of the professional associations somewhat specific to UK chiropractic, when compared with say law or medicine, is the number of professional associations that work in the “interest” of chiropractic and perhaps more significantly, compete for the relatively small number of chiropractors within the UK. Indeed, the survey results suggest that the BCA is not only the longest established of the UK chiropractic professional associations, but in the case of the WIOC Alumni, represents the majority (approximately 50 %) of respondents, far in advance of the other, younger UK chiropractic professional associations: namely the RCC (approximately 20 %) and UCA (approximately 15 %). This fits with presumed UK association membership figures taken from Fikar, Edlund and Newell [33].

Regarding those actual factors which might tip the decision regarding which association to join, ‘access to professional indemnity insurance’ rated the second most important factor at 89.2 %. Interestingly, just over a third (35.1 %) of respondents reported they would be willing to forgo membership of the existing chiropractic associations, if a suitable company providing indemnity insurance existed: it is worth mentioning that such companies do exist. Slightly in advance of this, at 91.2 %, was ‘promoting public awareness of chiropractic’. This concurs with the responses of a US school counsellors group study regarding factors affecting their choice to join or not join their professional organisations [23]. Questions asked included 1) ‘membership in professional organisations is one of the hallmarks of a professional person’ (results: 68.7 % agree: 31.3 disagree) and 2) ‘professional organisations increase the visibility of the profession’ (results: 89.2 % agree: 10.8 % disagree); showing a very similar proportional distribution to the results reported here.

Furthermore, despite the fact that the majority (88.8 %) of respondents were, upon graduation, a member of a UK chiropractic professional association, nearly one third (31.1 %) reported having actually resigned or more passively, not renewed membership of a UK chiropractic professional association for a variety of reasons. A similar study of pharmacy students reported comparable responses and response rates to discontinuing membership [5]. Reasons reported by pharmacy students for discontinuing membership included: lack of perceived benefit, cost, the organisation not aligning with his/her area of interest, unprofessional behaviour by organisations leadership, lack of organised activity by the organisation, and several logistical reasons echoing the results reported here.

Over and above differences of opinion, philosophy and identity [10–14], one further barrier to the unification of the four main UK chiropractic associations is in educational standards, namely, European Council of Chiropractic Education (ECCE) accreditation. The McTimoney College of Chiropractic (MCC) is not currently ECCE accredited and therefore MCC graduates cannot be members of the BCA [34]. However, the MCC did recently win its appeal against the decision of the ECCE Commission on Accreditation (CoA) not to accredit the program (referred to as crediting the college on their Facebook page [35]). The CoA resolved to commission a new evaluation report based on a new evaluation visit, however the MCC, as at the 16^th^ December 2015, is not willing to proceed further with this process [35].

In the course of this research project many themes/topics within chiropractic have been touched upon, including: professionalism, education, leadership, research, legislation, lobbying, unity, identity, philosophy and ethos, style of practice and prescribing rights. All are factors and motivations influencing joining (or not) UK chiropractic professional associations. It will be interesting to see over time, if unification of the four main UK chiropractic associations happens. Could these associations merge and sit under, for example the Royal College of Chiropractic (RCC) or alternatively, perhaps the Alliance of UK Chiropractors (AUKC) could serve as a vehicle. Such a unification may never come to pass, as differences in opinion and a range of viewpoints will probably continue to exist. Time will tell, however, the area is definitely in need of further research.

### Limitations of study

There are several limitations to this study. The potential impact of response and non-response bias must be considered. The response rates are suggestive of a trend: those who graduated in the early years since WIOC’s founding are not well represented, perhaps because they do not use Facebook and/or motivational issues [36]. Additionally, it is possible that alumni who are members of a UK chiropractic professional association will be more likely to complete the questionnaire than those who are not. Likewise, it may be that some professional associations have more vociferous/motivated members than others, and also similarly, those that answered could hold polarised views [36].

Furthermore, a significant limitation is the estimated 77.4 % non-response rate from ‘The WIOC Alumni’ Facebook group. Although the 22.6 % response rate does allow for conclusions to be drawn regarding the motivations and factors in the respondents, the WIOC Alumni is not represented in its entirety. This leads to less than robust confidence in the generalisation of the factors and motivations to the wider profession. Moreover, the results describe the (biased) sample.

Similarly, ‘The WIOC Alumni’ Facebook group membership is primarily comprised of WIOC graduates, but includes some final year WIOC students and other interested parties (who are not necessarily WIOC graduates). This has the potential outcome to skew the results.

Additionally, there are limits to generalising across any professional associations and indeed across UK chiropractic professional associations, which this study did. The term ‘professional association‘(for the purposes of this research) was intended as an umbrella term encompassing a variety of professional organisations ‘type’. This overarching use may have confused, raised bias and limited the results, especially when Association not preceded by ‘Professional’ within the field of UK chiropractic generally refers to the main four associations, BCA, MCA, SCA and UCA (who act like Trade Unions). Indeed, each UK chiropractic professional association is a unique entity, offering differing USPs, products, services and benefits, even though there are similarities. What is more, this research relied on published literature (not limited to the field of healthcare) with central themes of ‘professional organisations’ and ‘factors and motivations’ and what is applicable to one profession, or field, may not be specific enough for another [2]. It can be argued that there is strength in unity/numbers. There are a relatively small number of chiropractors within the UK, therefore, it appears sensible to suggest that unification of the four main chiropractic associations could positively impact on the professional development of the members, benefiting not only the organisation but aiding in advancing the profession as a whole. Regardless of the positives, unification may never come to pass, the differences in opinion and a range of viewpoints of a hard-line minority always exist. Further research is warranted to develop a greater understanding of this issue and to determine what might be best for the profession going forward.

The authors acknowledge limitations imposed by the administration and delivery of the survey instrument through the online platform. Disadvantages of an online survey instrument include the aforementioned: bias, survey fatigue, issues relating to ensuring representative sample and size, days and time of reminders; and privacy issues/anonymity. Such surveys are impersonal, and require very clear answer instructions. Additionally, respondents may experience frustration, particularly in relation to mandatory questions [27–30, 32, 36, 37]. Furthermore, Bristol Online Surveys (BOS) does not prevent a survey from being completed many times on the same computer or from the same IP address unless survey access control is utilised.

Survey design, formatting and question layout are of paramount importance. The final survey itself, contained minor typographical errors, which were not picked up during the validation stages. Additional to this and all questionnaires are those unavoidable limitations of the study design including: unexpected interpretational differences between respondents, perceived question ambiguity and even false reporting (conscious or subconscious/intentional or not) by the respondents [36].

### Recommendations

While being a snapshot of the current situation for those who responded, this survey could serve as a starting point for further work in this area. Future studies in this field will not only be of interest to the chiropractic profession, but potentially to other health care professionals as well.

Further research is required to determine if the results presented here are WIOC alumni specific or can be extrapolated more generally. It is also important to look more closely at what the UK chiropractic professional associations actually offer, along with further exploration of the perspectives, influences, motivations and other such factors.

Greater knowledge of the factors and motivations for membership can help UK chiropractic professional associations engage more efficiently with the wider profession as well as their own members and can help associations promote chiropractic, market, recruit and retain members more effectively. Although i is hoped in the future to open the survey to include the alumni of the other UK chiropractic educational institutions and the members of UK chiropractic professional associations, it would be interesting to determine which of these motivators are present internationally.

## Conclusions

Several factors have an important effect on motivations to join (or not) UK chiropractic professional associations. Those considered as strongly motivating to join included: ‘promoting public awareness of chiropractic’ (91.2 %), ‘access to professional indemnity insurance’ (89.2 %), ‘overall professionalism of the association’ (87.2 %), ‘the identity of the association’ (77.7 %), ‘positive attitude to research’ (77.0 %) and ‘workplace support and advice’ (68.9 %).

Future studies in this field will not only be of interest to the chiropractic profession, but potentially to other health care professionals as well. More research is required to determine how representative WIOC views are of the chiropractic profession as a whole both in the UK and further afield.

Unity is a theme in chiropractic, from this limited sample, the majority of respondents to this survey would support unification of the four main UK chiropractic associations, should this become an option sometime in the future.

## Abbreviations

AECC, Anglo European College of Chiropractic; AUKC, Alliance of UK Chiropractors; BACS, British Association of Chiropractic Students; BCA, British Chiropractic Association; BOS, Bristol Online Surveys; BVCA, British Veterinary Chiropractic Association; CoA, Commission on Accreditation; CPD, Continuing Professional Development; CRC, Chiropractic Research Council; EAC, European Academy of Chiropractic; ECCE, European Council on Chiropractic Education; FICS, The International Federation of Sports Chiropractic / Fédération Internationale de Chiropratique du Sport; GCC, General Chiropractic Council; MCA, McTimoney Chiropractic Association; MCC, McTimoney College of Chiropractic; RCC, Royal College of Chiropractors; SCA, Scottish Chiropractic Association; UCA, United Chiropractic Association; UK, United Kingdom; USPs, unique selling points; WIOC, Welsh Institute of Chiropractic

## Additional file

Additional file 1: The survey instrument used in this study. (DOCX 225 kb)
