# Supplementary material for: The factors and motivations behind United Kingdom chiropractic professional association membership: a survey of the Welsh Institute of Chiropractic Alumni
Source: Chiropr Man Therap. 2016 Sep 12;24(1):35. doi: 10.1186/s12998-016-0115-x (PMC5018935; doi:10.1186/s12998-016-0115-x)
Supplement: Additional file 1: — The survey instrument used in this study. (DOCX 225 kb) [file 12998_2016_115_MOESM1_ESM.docx]

To join or not to join? United Kingdom Chiropractic Professional Association Membership: Factors and Motivations

Page 1: Section 1: Welcome

You are invited to take part in a research study. Prior to participating it is important for you to understand why the research is being undertaken and what it will involve.

This research study aims to identify factors and motivations of UK chiropractic professional association membership, with the intention to develop a basic understanding of what members want from their chosen chiropractic professional association.

This questionnaire will take 5-10 minutes to complete and consists of 6 sections, totalling 23 questions.

Section 1: Welcome

Section 2: Introduction

Section 3: Demographics - Questions 1-9

Section 4: Memberships, Factors and Motivations - Questions 10-22

Section 5: Conclusion - Optional opportunity to comment on survey - Question

23

Section 6: Acknowledgement

2 / 19

All data will be collected and stored **anonymously** via Bristol Online Surveys

(BOS) or where applicable hard copy.

**No individuals will be identified in this research study.**

This research has been conducted **without** sponsorship from or affiliation to any chiropractic professional associations.

***Consent to take part is implied by completion and submission of***

***the survey.***

This study has been reviewed and approved by the Undergraduate Research

Module Review Group at the University of South Wales.

If you have any queries about any aspect of the research feel free to contact

Sheena Wotherspoon on: 11003189@students.southwales.ac.uk.

If you have a formal complaint, please contact the project supervisor, Prof Peter McCarthy, Faculty of Life Sciences and Education,

Innovation House, University of South Wales, CF37 1DL, on:

peter.mccarthy@southwales.ac.uk

3 / 19

Page 2: Section 2: Introduction

A professional association can be defined as a body of persons engaged in the same profession, formed usually to control entry into the profession, maintain standards, and represent the profession in discussions with other bodies.

Within the UK chiropractic profession, there are multiple professional associations representing chiropractors. This research focuses on professional membership bodies such as the Royal College of Chiropractic, research bodies, such as the Chiropractic Research Council and the Associations which function to facilitate chiropractors and chiropractic.

In this instance, chiropractic educational institutions, their specific alumni/graduate associations, and the General Chiropractic Council (GCC) are intentionally omitted from the questionnaire listings regarding memberships, factors and motivations (Section 4). This is because in order to practice as a chiropractor within the UK, one must be registered with the GCC, and, one must have studied at a chiropractic educational institution. It is assumed that on graduation from an educational institution one automatically receives ‘alumni’ status from that educational institution, whether one officially joins, or does not join, said alumnus.

4 / 19

Page 3: Section 3: Demographics

These questions are being asked to ensure that the inclusion criteria for this study is met.

*1* Are you:  *Required*

Male Female Prefer not to say

*2* What is your age?  *Required*

18 to 24 25 to 34 35 to 44

45 to 54 55 to 64 65 to 74

75 or older Prefer not to answer

*3* Which institution did or will you graduate from?  *Required*

Anglo European College of Chiropractic (AECC)

McTimoney College of Chiropractic (MCC)

Welsh Institute of Chiropractic (WIOC)

Other

*3.a* If you selected Other, please specify:

5 / 19

*4* When did or will you graduate as a chiropractor? Please select the year. 

*Required*

*5* Are you currently practicing as a registered chiropractor in the UK? 

*Required*

Registered: Practicing

Registered: Not practicing

Not registered: Practicing

Not registered: Not practicing

6 / 19

Page 4: Section 3: Demographics Continued

*6* Were you an EU or international student? (i.e. your country of origin is outside the United Kingdom which is comprised of England, Scotland, Wales and Northern Ireland).  *Required*

Yes No

Please select between 1 and 6 answers.

*7* Please tick all that apply. Are you:  *Required*

A sole practitioner

A practice owner

An associate

Working full time

Working part time

A student

Any other (e.g. on a career break)

Foundation Year Year 1 Year 2

Year 3 Year 4 Year 5

*7.a* If you selected “A student”, please select your year of study:

*7.b* If you selected “Any other (e.g. on a career break)”, please specify:

7 / 19

*8* Are you a member of faculty, or staff, or employed, at the Anglo European College of Chiropractic, McTimoney College of Chiropractic or the University of South Wales?  *Required*

Yes No

*8.a* If you answered Yes in Question 8, are you:

Full time Part time Hourly paid Other

*8.a.i* If you selected Other, please specify:

*9* Are you employed by or actively involved in any of the UK chiropractic professional associations (including, for example: holding office, serving on committees)?  *Required*

Yes No

8 / 19

Page 5: Section 4: Memberships, Factors and

Motivations

*10* Have you ever held a position of leadership within any professional association? This can be in any field and anywhere in the world. For example, a student society committee membership would count as a Yes.  *Required*

Yes No

*11* Do you agree or disagree that membership in any professional association is one of the hallmarks of a professional person?  *Required*

Agree Disagree

*11.a* If you would like to comment or explain your answer further please do so

here: *Optional*

*12* Were you ever a student member of any of the following UK chiropractic professional associations? Please tick all that apply.  *Required*

Please select between 1 and 6 answers.

British Chiropractic Association (BCA)

McTimoney Chiropractic Association (MCA)

Scottish Chiropractic Association (SCA)

United Chiropractic Association (UCA)

Royal College of Chiropractors (RCC) (formerly College of Chiropractic)

Other(s)

I have never been a student member of any of the UK chiropractic

professional associations

*12.a* If you selected Other(s), please add and specify:

*12.b* If you would like to comment or explain your answer(s) further please do

so here: *Optional*

*13* On graduation as a chiropractor, which UK professional chiropractic association did or will you join? You can select more than one box.  *Required*

Please select between 1 and 10 answers.

I am not currently a member of any UK professional chiropractic association

I have not yet decided

I did not or will not join any UK professional chiropractic association

British Chiropractic Association (BCA)

McTimoney Chiropractic Association (MCA)

Scottish Chiropractic Association (SCA)

United Chiropractic Association (UCA)

Royal College of Chiropractors (RCC) (formerly College of Chiropractic)

Chiropractic Research Council (CRC)

Other(s)

10 / 19

*13.a* If you selected Other(s), please add and specify:

*13.b* If you would like to comment or explain your answer(s) further please do so here:

*14* Excluding membership as a student, have you ever resigned, or not renewed, membership of any UK professional chiropractic association? 

*Required*

Yes No

*14.a* If you answered Yes to Question 14, please state your reason for resigning, or not renewing, membership.

*15* Excluding membership as a student, how many years have you been a member of a UK professional chiropractic association?  *Required*

I am not a member

Less than 1 year

1-5 years

6-10 years

11-15 years

16+ years

Page 6: Section 4: Memberships, Factors and

Motivations Continued

**

**

*16.a* If you would like to comment or explain your answer(s) further please do so here: *Optional*

*17* Please add any factors that you feel are missing from the listing in Question 16. *Optional*

Page 7: Section 4: Memberships, Factors and

Motivations Continued

*18* Do you agree or disagree that chiropractic professional associations increase the visibility of the chiropractic profession within the UK?  *Required*

Agree Disagree

*20* Would you want others to know you are a member of a chiropractic professional association?  *Required*

Yes No

*20.a* If you would like to comment or explain you answer further, please do so here: *Optional*

*21* Would you support, should it become an option sometime in the future, the unification of the four main UK chiropractic associations (BCA, MCA, SCA, UCA)?  *Required*

Yes No

*21.a* If you would like to comment or explain you answer further, please do so here: *Optional*

*22* If there is a suitable company providing indemnity insurance, would you forgo membership of the existing UK chiropractic associations?  *Required*

Yes No

*22.a* If you would like to comment or explain you answer further, please do so here: *Optional*
